# mot-2–Mediated Cross Talk between Nuclear Factor-κB and p53 Is Involved in Arsenite-Induced Tumorigenesis of Human Embryo Lung Fibroblast Cells

**DOI:** 10.1289/ehp.0901677

**Published:** 2010-03-03

**Authors:** Yuan Li, Yuan Xu, Min Ling, Ye Yang, Shoulin Wang, Zhong Li, Jianwei Zhou, Xinru Wang, Qizhan Liu

**Affiliations:** Department of Molecular Cell Biology and Toxicology, School of Public Health, Nanjing Medical University, Nanjing, Jiangsu, People’s Republic of China

**Keywords:** arsenite, mot-2, NF-κB, p53, signal transduction, tumorigenesis

## Abstract

**Background:**

Inactivation of p53 is involved in arsenite-induced tumorigenesis; however, the molecular mechanisms remain poorly understood.

**Objective:**

We investigated the molecular mechanisms underlying the inactivation of p53 and neoplastic transformation induced by arsenite in human embryo lung fibroblast (HELF) cells.

**Methods:**

Anchorage-independent growth assays were performed, and tumorigenicity in intact animals was assessed to confirm arsenite-induced neoplastic transformation. We determined the levels and functions of p53, nuclear factor-kappa B (NF-κB; a key transcriptional regulator), and mot-2 (a p53 inhibitor) and their relationships in arsenite-induced transformed HELF cells by two-dimensional electrophoresis, reverse-transcriptase polymerase chain reaction, Western blot, immunofluorescence, and co-immunoprecipitation assays.

**Results:**

Exposure of HELF cells to low levels of arsenite increased their proliferation rate and anchorage-independent growth and disrupted normal contact inhibition. When introduced into nude mice, transformed cells were tumorigenic. We used proteomic analysis to identify proteins with altered expression between untreated and arsenite-exposed cells. We found decreased expression of NF-κB repressing factor (NKRF; an inhibitor of NF-κB–mediated gene transcription), increased expression of mot-2, and increased activation of NF-κB. Changes in cells exposed to 1.0 μM arsenite were more marked than changes in cells exposed to 0.5 or 2.0 μM arsenite. Inactivation of NF-κB prevented malignant transformation induced by 1.0 μM arsenite. Moreover, we also identified a mechanism whereby NF-κB regulated p53. Specifically, activation of NF*-*κB up-regulated *mot-2* expression, which prevented nuclear translocation of p53 and switched the binding preference of the p53 and NF-κB coactivator CBP [cyclic AMP-responsive element binding protein (CREB) binding protein] from p53 to NF-κB.

**Conclusions:**

mot-2–mediated cross talk between NF-κB and p53 appears to be involved in arsenite-induced tumorigenesis of HELF cells.

Arsenite is an environmental toxicant that has been associated with numerous human health problems, including dermatosis, diabetes mellitus, cardiovascular disorders, and cancer (Tapio and Grosche 2006). Epidemiologic evidence implicates exposure to arsenite in causing human cancers of the skin, lung, and bladder [International Agency for Research on Cancer (IARC) 2004]. Although skin is thought to be the most sensitive site for arsenite toxicity, the lung is now recognized as a specific target (Chen et al. 2004). *In vitro*, arsenite causes malignant transformation of various cells, including human keratinocytes and small airway epithelial cells (Pi et al. 2008; Wen et al. 2008). Arsenite induces tumorigenesis by activating signal pathways and transcription factors, such as mitogen-activated protein kinases, activating factor-1, and nuclear factor-κB (NF-κB), which are involved in promoting cell proliferation and malignant transformation (Huang et al. 2004; Salnikow and Zhitkovich 2008). Although arsenite is carcinogenic, the mechanisms involved remain unclear.

In mammalian cells, p53 (often called the “guardian of the genome”) is involved in the maintenance of genome stability. In response to diverse cellular stresses, p53 protein transactivates downstream target genes required for DNA repair, cell cycle arrest, and apoptosis (Pietsch et al. 2008). People carrying one dysfunctional p53 gene in their germline have a high probability of developing a tumor. Restoration of p53 function inhibits ultraviolet B–induced skin carcinogenesis. Thus, functional inactivation of p53 is a common characteristic of tumors (Konstantakou et al. 2009; Lim and Park 2009). Involved in the inactivation of p53 are the following mechanisms: *a*) mutations of *p53* that eliminate its function in DNA binding or transcriptional activation; *b*) abnormal expression of p53-interacting proteins (e.g., mdm2), which results in accelerated degradation of wild-type p53 or in stabilization of mutant p53; and *c*) nuclear exclusion of wild-type p53 (Huang et al. 2008; Wadhwa et al. 2002). Although the first two categories have been investigated intensively, the third remains poorly understood.

Data relating to the effects of arsenite on p53 are conflicting. Although high levels of arsenite generally induce an increase in p53 activity in response to arsenite-induced DNA damage, lower levels yield results that are dependent on the compound, concentration, duration of treatment, and cell type (Bodwell et al. 2004; Pi et al. 2005). Apparently, the tumor suppressor function of p53 must be compromised before a cell undergoes arsenite-mediated transformation (Aylon and Oren 2007; Kastan 2007). p53 dysfunction is necessary for arsenite-induced centrosome abnormalities and transformation in human lung cells (Liao et al. 2007). Defining the molecular mechanism of p53 inactivation in arsenite-induced neoplastic transformation of cells is necessary for a complete understanding of the oncogenesis caused by arsenite. In the present study, we exposed human embryo lung fibroblast (HELF) cells to low levels of arsenite [see Supplemental Material, Figure 1A (doi:10.1289/ehp.0901677)] to investigate underlying mechanisms of arsenic-mediated effects on p53 function and neoplastic transformation of these cells.

## Materials and Methods

### Cells, cell culture, and arsenite exposure

The HELF cell line and A549 cell line were obtained from the Shanghai Institute of Cell Biology, Chinese Academy of Sciences (Shanghai, China). HELF cells are human sarcoma virus-40 (SV-40) immortalized and nontumorigenic diploid fibroblasts from lungs of hysterotomy-derived embryos (Hayflick and Moorhead 1961). HELF cells have normal p53 function and signal pathways and are used as a model of lung damage and neoplastic transformation induced by environmental agents (Gao et al. 2007; Jiao et al. 2008; Wang et al. 2005). A549 cells, human lung adenocarcinoma epithelial cells, are often used as positive controls in lung cancer research. Cells were cultured as described previously (He et al. 2007). For chronic exposure, 1 × 10^6^ cells were seeded into 10-cm (diameter) dishes for 24 hr and maintained in 0.0, 0.5, 1.0, or 2.0 μM sodium arsenite (NaAsO_2_; Sigma, St. Louis, MO, USA; 99.0% purity) for 48–72 hr per passage. This process was continued for about 15 weeks (30 passages). The NF-κB inhibitor Bay11-7082 (Sigma) was dissolved in dimethyl sulfoxide (Sigma). All other reagents used were of analytical grade or the highest grade available.

### Serum starvation

We routinely cultured 2 × 10^4^ cells in Dulbecco’s modified Eagle’s medium (DMEM) supplemented with 10% fetal bovine serum (FBS) in six-well plates for 24 hr. For serum starvation, the medium was changed to 1% FBS. After 96 hr, the cells, in triplicate, were collected and counted under a microscope.

### Anchorage-independent growth

Soft agar dishes were prepared with underlayers of 0.70% agarose in DMEM supplemented with 10% FBS. To test for soft-agar colony growth capacity, cells were plated in triplicate at a density of 1 × 10^4^ in 2 mL 0.35% agarose over the agar base. Cultures were fed every 3 days; after 14 days, colonies with > 30 cells were examined microscopically.

### Tumorigenicity in intact animals

This study was performed according to a protocol approved by the Nanjing Medical University Institutional Animal Care and Use Committee, and animals were treated humanely and with regard for alleviation of suffering. Briefly, 1 × 10^7^ cells were injected subcutaneously into the right armpit of nude BALB/c mice 6 (six mice per group). Four weeks later, the tumor tissues were removed, fixed with 4% formalin, embedded in paraffin, sectioned, stained with hematoxylin and eosin, and analyzed by light microscopy.

### Analysis of cellular protein level alterations

Differences in levels of cellular proteins between passage control cells and transformed cells were detected by two-dimensional electrophoresis (2DE). Detection of protein spots, in-gel tryptic digestion, mass spectrometry, and statistical analysis were performed as described previously (Zhang et al. 2007).

### RNA interference

We purchased control small interfering RNA (siRNA), *RelA* siRNA, and *p53* siRNA from Cell Signaling Technology (Beverly, MA, USA). The oligonucleotides for *mot-2* siRNA were 5′-GGAUUGUCACUGAUCUAAU-3′ and 5′-AUUAGAUCAGUGACAAUCC-3′ (Sigma). We performed cell transfections using the N-TER Nanoparticle siRNA Transfection System (Sigma). Briefly, 7 × 10^5^ cells were seeded into each well of six-well plates, 18–24 hr before transfection. Nanoparticle formation solution containing 20 nM target gene siRNA was added to transfection medium and transferred to each well of the culture plates. After 24 hr, cells were harvested for Western blot, co-immunoprecipitation, or immunostaining assays.

### Reverse-transcriptase polymerase chain reaction (RT-PCR)

Total RNA (2 μg) was transcribed into cDNA using AMV Reverse Transcriptase (Promega, Madison, WI, USA). We used *mot-2* primers (forward, 5′-CGAGTCAGATTGGAGCAT-3′; reverse, 5′-GACCATAGGCAAGAGCAG-3′) for PCR amplification.

### Immunostaining

Treated cells were incubated with rabbit phospho-p53 (p-p53) antibody (Cell Signaling Technology) at 4°C overnight and then incubated with Cy3-conjugated goat anti-rabbit secondary antibody (Millipore, Billerica, MA, USA) for 1 hr. The nuclei were stained by adding 4′,6-diamidino-2-phenylindole (DAPI; Sigma) for 10 min. The cells were observed under a fluorescence microscope (Olympus, Tokyo, Japan). We analyzed fluorescence intensities using a multimode microplate reader (Tecan Trading AG, Männedorf, Switzerland) and images with an Image-Pro Plus 6.0 (Olympus).

### Western blots

Cell lysates were separated by SDS-PAGE and transferred to polyvinylidene fluoride membranes (Millipore); the immune complexes were detected by enhanced chemiluminescence (Cell Signaling Technology). We used the following antibodies: NF-κB repressing factor (NKRF), CBP [cyclic AMP responsive element binding protein (CREB) binding protein], mot-2 (a p53 inhibitor), and β-actin (all from Sigma); and NF-κB inhibitor (IκBα), phosphorylated IκBα [p-IκBα (serine 32)], RelA (a subunit of NF-κB), phosphorylated RelA (p-RelA; serine 536), wild-type p53, p-p53 (serine 15), and proliferating cell nuclear antigen (PCNA) (all from Cell Signaling Technology). Blots were quantitated by densitometry and normalized using β-actin to correct for differences in protein loading. For densitometric analyses, we measured protein bands on the blot using Eagle Eye II software (He et al. 2007).

### Co-immunoprecipitation

Cells were extracted for 30 min with lysis buffer. After centrifugation of the preparations, the supernatants were incubated with p53 or CBP antibody and subsequently with A+G Sepharose beads (Sigma) at 4°C overnight. The pellets were washed three times, resuspended in the SDS sample buffer, and boiled to remove protein from the beads. The immunoprecipitants were analyzed by Western blots with mot-2, RelA, or p53 antibodies.

### Statistical analysis

All numeral data, except tumor incidence and tumor volumes, were generated from three independent experiments and expressed as mean ± SD. We used one-way analysis of variance (ANOVA) to assess significant differences among groups. Statistical significance, determined by the Fisher test, was set at *p <* 0.05.

## Results

### Neoplastic transformation of HELF cells induced by arsenite

**Figure 1:**
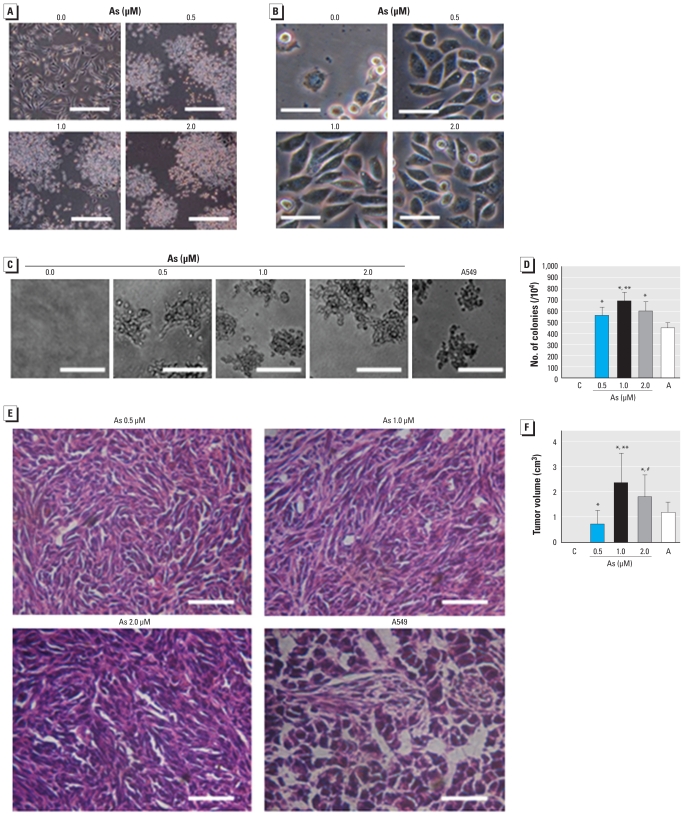
Neoplastic transformation of HELF cells exposed to 0.0, 0.5, 1.0, or 2.0 μM arsenite (As) for about 15 weeks (30 passages). C, control (untreated) HELF cells. A549 carcinoma cells served as the positive control. Morphological images of cells (*A*) after culture with 10% FBS (bars = 500 μm) and (*B*) after culture with 1% FBS (bars = 50 μm). (*C*) Photomicrographs of cell colonies in soft agar; bars = 500 μm. (*D*) The number (mean ± SD) of cell colonies in soft agar (*n* = 3). (*E*) Representative pathological sections of tumors 4 weeks after cells were inoculated into nude/BALB/c mice; tumors induced by arsenite-transformed cells consisted of undifferentiated and spindle cells (bars = 100 μm). (*F*) Volume (mean ± SD) of tumors in nude/BALB/c mice (*n* = 6). *p < 0.01 compared with control group. **p < 0.05 compared with 0.5 or 2.0 μM arsenite groups. ^#^*p* < 0.05 compared with mice implanted with cells exposed to 0.5 μM arsenite.

To evaluate oncogenic transformation, we exposed HELF cells to 0.0, 0.5, 1.0, or 2.0 μM arsenite. After 15 weeks, the passage control cells grew in a monolayer, showed the typical elongated shape of fibroblast-like cells, and stopped dividing after reaching confluence. In contrast, transformed cells showed an epithelial-like morphology; after reaching confluence, they grew in multilayers and formed cellular aggregates (Figure 1A). The doubling time of the passage control cells was 27.6 ± 2.1 hr; the values for transformed cells induced by 0.5, 1.0, and 2.0 μM arsenite were 22.8 ± 2.6, 22.3 ± 2.1, and 22.7 ± 1.7 hr, respectively. The levels of PCNA in transformed cells were higher than in passage control cells [see Supplemental Material, Figure 1B (doi:10.1289/ehp.0901677)]. Moreover, transformed cells tolerated low levels of serum (Figure 1B). In agar, 552 ± 84, 689 ± 71, and 598 ± 83 colonies were formed in HELF cells exposed to 0.5, 1.0, and 2.0 μM arsenite, respectively, and 449 ± 43 colonies were formed in A549 cells. In contrast, passage control cells showed no anchorage-independent growth (Figure 1C,D).

In the nude BALB/c mice injected with HELF cells exposed to 0.5, 1.0, and 2.0 μM arsenite and in the positive control mice injected with A549 carcinoma cells, tumor incidences were all 100% (6 of 6 per group); for the passage control group, however, the incidence was 0% (0 of 6). Tumors induced by arsenite-transformed cells consisted of either highly undifferentiated or spindle cells. The tumor volumes for the groups implanted with cells exposed to 0.5, 1.0, and 2.0 μM arsenite were 0.73 ± 0.59, 2.29 ± 1.23, and 1.79 ± 0.98 cm^3^, respectively, and 1.26 ± 0.31 cm^3^ for the A549 positive-control group (Figure 1E,F).

### Identification of proteins differentially expressed in arsenite-induced transformed HELF cells

**Figure 2:**
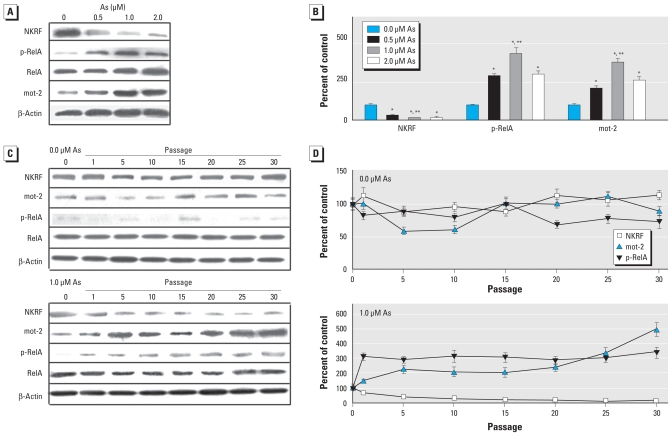
The levels of NKRF and mot-2 and the activation of NF*-*κB in HELF cells transformed by arsenite (As). β-Actin levels, measured in parallel, served as controls. (*A*) Levels of NKRF, p-RelA, RelA, and mot-2 in HELF cells exposed to 0.0, 0.5, 1.0, or 2.0 μM arsenite for about 15 weeks (30 passages) as detected by Western blots. (*B*) Relative protein levels (mean ± SD) of NKRF, p-RelA, and mot-2 (*n* = 3). (*C*) Western blot analyses of NKRF, mot-2, p-RelA, and RelA in HELF cells exposed to 0.0 μM (top) or 1.0 μM (bottom) arsenite for 1, 5, 10, 15, 20, 25, or 30 passages. (*D*) Relative protein levels (mean ± SD) of NKRF, p-RelA, and mot-2 in passage control cells (top) and in cells exposed to 1.0 μM arsenite (bottom); *n* = 3. *p < 0.01 compared with passage control cells. **p < 0.05 compared with cells exposed to 0.5 or 2.0 μM arsenite.

Of 5,671 protein spots, 126 were different between the passage control cells and transformed cells [see Supplemental Material, Figure 2A,B (doi:10.1289/ehp.0901677)]. Of these, we selected NKRF (an NF-κB inhibitor) and mot-2 (a p53 inhibitor) for further analysis. The NKRF in transformed cells was 73% lower than in passage control cells; mot-2 in transformed cells was 110% higher than in passage control cells [see Supplemental Material, Figure 2C (doi:10.1289/ehp.0901677)]. Verifying the results of 2DE, Western blots confirmed the down-expression of NKRF and up-expression of mot-2 in transformed cells. However, these changes in the 1.0-μM arsenite group were more marked than those for the 0.5- or 2.0-μM arsenite groups. Further, p-RelA, which indicates the activation of NF-κB, was higher in transformed cells than in passage control cells (Figure 2A,B). With increased time of exposure to arsenite, we found more malignant cells and greater down-expression of NKRF and up-expression of p-RelA and mot-2. No such changes, however, were observed in passage control cells (Figure 2C,D).

### NF-κB activation as a regulator of mot-2 in arsenite-exposed HELF cells

**Figure 3:**
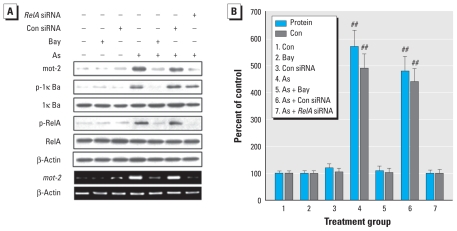
Effects of blocking NF-κB on mot-2 protein and mRNA levels and *NF*-κ*B* activation in HELF cells exposed to arsenite (As). HELF cells were exposed to 10 μM Bay11-7082 (Bay) for 3 hr or to 20 nM control (Con) siRNA or *RelA* siRNA for 24 hr, then incubated with 0.0 or 1.0 μM arsenite for 3 hr (p-IκBα and p-RelA) or 24 hr (mot-2, IκBα, and RelA). β-Actin levels, measured in parallel, served as controls. (*A*) Western blot analyses of mot-2, p-IκBα, IκBα, p-RelA, and RelA (top), and RT-PCR analyses of *mot-2* (bottom). (*B*) Relative protein or mRNA levels of mot-2 (mean ± SD; *n* = 3). ^##^*p* < 0.01 compared with cells exposed to arsenite plus Bay11-7082 or arsenite plus *RelA* siRNA.

Based on the present results, which showed NF-κB activation and accompanying increases of mot-2 during arsenite-induced neoplastic transformation, and the fact that the sequence GGAGGTTTCC of the *mot-2* promoter is similar to *kappaB* DNA elements (GGGRNYYYCC) [see Supplemental Material, Figure 3A (doi:10.1289/ehp.0901677)], we investigated the possibility that the mot-2 increases were caused by *NF-*κ*B* activation. The levels of p-IκBα, p-RelA, and mot-2 were elevated by 1.0 μM arsenite. However, inhibition of *NF-*κ*B* by Bay11-7082 [a compound that blocks IκBα phosphorylation and consequently prevents nuclear translocation of the activated NF-κB complex; see Supplemental Material, Figure 3B,C (doi:10.1289/ehp.0901677)] or *RelA* siRNA (which inhibits translation of the RelA NF-κB subunit) blocked the arsenite-induced increases of mot-2 protein and mRNA levels (Figure 3A,B).

### Involvement of NF-κB and mot-2 in the activation and translocation of p53 in arsenite-treated HELF cells

**Figure 4:**
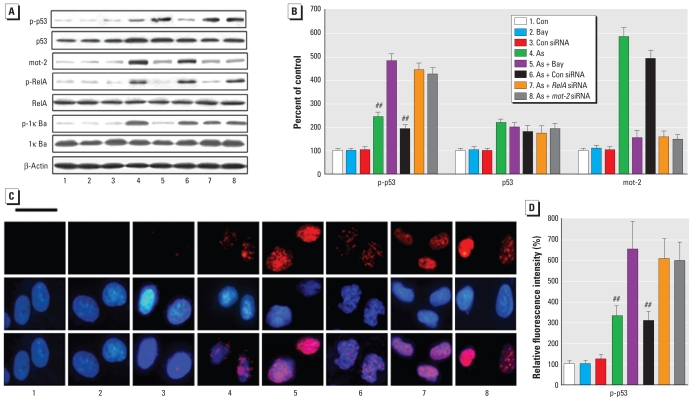
Involvement of NF-κB and mot-2 in the activation and translocation of p53 in HELF cells exposed to arsenite (As). HELF cells were exposed to 10 μM Bay11-7082 (Bay) for 3 hr or to 20 nM control (Con) siRNA, *RelA* siRNA, or *mot-2* siRNA for 24 hr, then incubated with 0.0 or 1.0 μM arsenite for 3 hr (p-p53, p-IκBα, and p-RelA) or 24 hr (p53, mot-2, RelA, and IκBα); fluorescence intensities were analyzed with a multimode microplate reader. β-Actin levels, measured in parallel, served as controls. (*A*) Western blot analyses of p-p53, p53, mot-2, p-RelA, RelA, p-IκBα, and IκBα. (*B*) Relative protein levels (mean ± SD) of p-p53, p53, and mot-2 (*n* = 3). (*C*) Immunofluorescence staining for p53 localization in HELF cells: blue, DAPI staining for nuclear DNA; red, p-p53. Bar, 25 μm. (*D*) Relative intensity (mean ± SD) of nuclear p53 fluorescence (*n* = 3). ^##^*p* < 0.05 compared with cells exposed to arsenite plus Bay11-7082, arsenite plus *RelA* siRNA, or arsenite plus *mot-2* siRNA.

Because mot-2 negatively regulates p53 activation (Wadhwa et al. 2002) and because NF-κB activation enhances *mot-2* expression (Figure 3), there could be mot-2–mediated cross talk between NF-κB and p53 in arsenite-exposed HELF cells. Blockage of *NF-*κ*B* (by Bay11-7082 or *Rel-A* siRNA) or *mot-2* (by *mot-2* siRNA) further increased the 1.0 μM arsenite-induced elevation of p-p53 but not of p53, which indicated arsenite-induced elevation of nuclear p53 (p-p53) but not of cytosolic (transcriptionally inactive) p53 (Figure 4A,B). In parallel with these results, inhibition of either *NF-*κ*B* or *mot-2* increased the nuclear translocation of p53 induced by 1.0 μM arsenite (Figure 4C,D).

### mot-2–mediated cross talk between NF-κB and p53 in arsenite-exposed HELF cells

**Figure 5:**
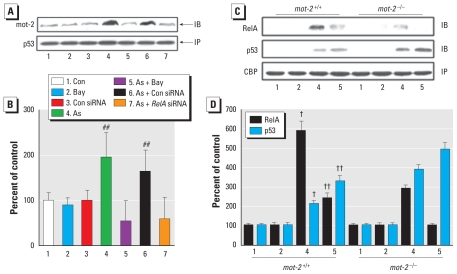
mot-2–mediated cross talk between *NF-*κ*B* and *p53* in HELF cells exposed to arsenite (As). (*A*) Western blot analyses of mot-2 and p53 after cell lysates were subjected to co-immunoprecipitation with p53 (IP) and mot-2 (IB) antibodies. HELF cells were exposed to 10 μM Bay11-7082 for 3 hr or to 20 nM control (Con) siRNA or *RelA* siRNA for 24 hr, then incubated with 0.0 or 1.0 μM arsenite for 24 hr. (*B*) Relative protein levels (mean ± SD) of mot-2 (*n* = 3). (*C*) Western blot analyses of RelA, p53, and CBP after cell lysates were subjected to co-immunoprecipitation with CBP (IP) and RelA or p53 (IB) antibodies. After *mot-2*–normal or *mot-2*–defective HELF cells were exposed to 10 μM Bay11-7082 for 3 hr, they were incubated with 0.0 or 1.0 μM arsenite for 24 hr. (*D*) Relative protein levels of RelA and p53 (mean ± SD; *n* = 3). ^##^*p* < 0.05 compared with cells exposed to arsenite plus Bay11-7082 or arsenite plus *RelA* siRNA. ^†^*p* < 0.05 compared with corresponding arsenite exposure in *mot-2*–knockdown cells. ^††^*p* < 0.05 compared with corresponding arsenite and Bay11-7082 exposure in *mot-2*–knockdown cells.

Arsenite increased the binding of mot-2 to p53, which was attenuated by blocking of NF-κB activity (Figure 5A,B). Furthermore, in *mot-2*–normal cells with a blocked NF-κB signal pathway, 1.0 μM arsenite promoted binding of the CBP coactivator to p53 instead of NF-κB; the change, however, was not evident in *mot-2*–knockdown cells (Figure 5C,D).

### Role of NF-κB in arsenite-induced neoplastic transformation of HELF cells

**Figure 6:**
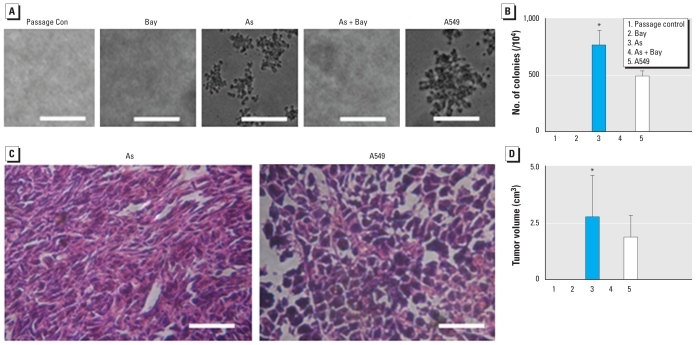
Involvement of NF-κB in arsenite-induced tumorigenesis in HELF cells exposed for about 15 weeks (30 passages) to 0.0 or 1.0 μM arsenite (As), to 10 μM Bay11-7082 (Bay), or to arsenite plus Bay11-7082. A549 cells served as the positive control (Con). (*A*) Cell colonies in soft agar; bars = 500 μm. (*B*) Number of cell colonies in soft agar (mean ± SD; *n* = 3). (*C*) Representative pathological sections of tumors 4 weeks after cells were inoculated into nude/BALB/c mice. Tumors induced by arsenite-transformed cells were composed of undifferentiated and spindle cells; bars = 100 μm. (*D*) Volume (mean ± SD) of tumors in nude/BALB/c mice (*n* = 6). *p < 0.01 compared with control cells, Bay11-7082 cells, or arsenite plus Bay11-7082 cells.

During transformation, Bay11-7082 effectively blocked arsenite-induced NF-κB activation [see Supplemental Material, Figure 4 (doi:10.1289/ehp.0901677)]. After 15 weeks of culture in agar, 724 ± 106 and 496 ± 41 colonies were formed from HELF cells exposed to 1.0 μM arsenite and from A549 carcinoma cells, respectively. In contrast, unexposed HELF cells and HELF cells exposed to Bay11-7082 with or without 1.0 μM arsenite showed no anchorage- independent growth (Figure 6A,B). In mice injected with arsenite–exposed cells (1.0 μM) and mice injected with A549 carcinoma cells, tumor incidences were 100% (6 of 6 per group); tumor incidences for the other three groups were 0% (0 of 6 per group). Tumor volumes were 2.63 ± 1.99 cm^3^ and 1.76 ± 0.96 cm^3^ in animals implanted with HELF cells exposed to 1.0 μM arsenite or with A549 cells respectively (Figure 6C,D).

### No mutations of p53 in neoplastic transformation of HELF cells induced by arsenite

In transformed HELF cells, the DNA sequences of exons 1–10 were a 100% match to *p53* cDNA after DNA fragments including exons 1–10 of *p53* were sequenced using primers of PCR amplification [see Supplemental Material, Figure 5 (doi:10.1289/ehp.0901677)].

## Discussion

Inorganic arsenite is a widely distributed, naturally occurring environmental contaminant affecting tens of millions of people worldwide (IARC 2004). Chronic exposure to low levels of arsenite (< 5 μM) usually causes cell proliferation, which leads to neoplastic transformation, whereas high levels cause cytotoxicity, indicating that the cellular effects of arsenite are dependent on the degree of exposure (Shen et al. 2008; Wen et al. 2008). In the present study, repeated exposure of HELF cells to 0.5, 1.0, or 2.0 μM arsenite resulted in neoplastic transformation of these cells, as determined by anchorage-independent growth in soft agar and tumorigenesis in nude mice.

In the United States and China, the current maximum contaminant level for arsenite in drinking water is 10 μg/L (~ 0.5 μM) (Huang et al. 2008). Therefore, in this study, we used 0.5, 1.0, and 2.0 μM concentrations of arsenite for long-term exposure of HELF cells. These levels of arsenite do not cause cell death and are lower than levels in drinking water in areas where arsenicosis is common (Pi et al. 2002; Tokar et al. 2010). Further, for cells exposed to 1.0 μM arsenite, the numbers of colonies and the volumes of tumors that developed in nude mice were higher than corresponding values for the 0.5 and 2.0 μM arsenite-exposed cells; this is in accord with the cell proliferation rate induced by 1.0 μM arsenite, which was higher than that induced by 0.5 or 2.0 μM arsenite. Therefore, the neoplastic transformation of HELF cells induced by low levels of arsenite is apparently associated with cell proliferation.

For this study, we selected HELF cells for assessing the p53 response to low levels of arsenite because these cells have a normal p53 function and normal signal pathways and are used as a model of lung damage and neoplastic transformation induced by environmental agents (Gao et al. 2007; Jiao et al. 2008; Wang et al. 2005). Biomethylation activates arsenite as a toxin and a carcinogen (Kojima et al. 2006; Sakurai et al. 2006). Because the capacity of HELF cells for arsenite biomethylation is unknown, the transformative agent in this study could have been inorganic arsenite, a methylated metabolite, or a combination of inorganic and methylated metabolites.

Interference by arsenite of the activation of p53 via poly-ADP-ribosylation may be involved in its comutagenic and cocarcinogenic effects (Yu et al. 2008). Because in this study we found no mutations of *p53* but did observe disruption of p53 function in transformed HELF cells, the low levels of arsenite apparently induced the dysfunction of p53 via a signal pathway.

NF-κB activation is thought to initiate and accelerate tumorigenesis, and its inhibition blocks cell transformation induced by tumor promoters (Li et al. 1997; Perkins 2007). Reactive oxygen stress, induced by low concentrations of arsenite (< 5 μM), activates NF-κB (Wijeweera et al. 2001). Data in the present study show that low levels of arsenite increase IκBα phosphorylation (thereby promoting nuclear translocation of NF-κB) and RelA phosphorylation, indicating that activation of NF-κB is accomplished by classical signaling pathways in arsenite-induced transformation.

*mot*-*2*, which is expressed in many cell types and tissues, possesses diverse activities, including oncogenic transformation. Moreover, many human transformed and tumor-derived cells have high *mot-2* expression (Wadhwa et al. 1999, 2002). mot-2 inactivates p53 function by directly binding to its C-terminal amino acid residues (Kaul et al. 2005). In human cells, mot-2 levels are increased by ionizing radiation and ozone (Wu et al. 1999). However, the mechanisms by which mot-2 exerts its effects remain unclear. In the present study, low levels of arsenite caused increases of *mot-2* mRNA and protein levels, but this effect was not evident for cells in which NF-κB activation was blocked. This indicates that the NF-κB signal pathway is involved in *mot-2* expression in arsenite-exposed HELF cells. The data also indicate that *mot-2*, regulated by NF-κB activation, was involved in the cross talk between NF-κB and p53 in arsenite-transformed HELF cells. In contrast, decreases in NKRF, increases in p-RelA and mot-2, and increases in cell colony numbers in soft agar and tumor volumes in nude mice induced by 1.0 μM arsenite were more pronounced than those caused by 0.5 or 2.0 μM arsenite. Moreover, blocking of NF-κB in HELF cells prevented the increase in mot-2, nuclear translocation of p53, and neoplastic transformation induced by 1.0 μM arsenite.

Cross talk between NF-κB and p53 negatively regulates their activities by competing for limiting pools of the p300 and CBP coactivators, which are required by both NF-κB and p53 for transactivation (Webster and Perkins 1999). IKKα-mediated phosphorylation of CBP switches the protein-binding preference of CBP from p53 to NF-κB and results in concurrent up-regulation of NF-κB–mediated genes and down-regulation of p53-mediated genes; this leads the cell toward tumorigenesis (Huang et al. 2007). The present results also show that, in *mot-2*–normal cells with a blocked NF-κB signal pathway, 1.0 μM arsenite increased CBP binding to p53 instead of binding to NF-κB; the change, however, was not evident in *mot*-*2*–knockdown cells.

## Conclusion

In arsenite-induced neoplastic transformation of HELF cells, activation of NF-κB increases *mot-2* expression by regulating its transcription. As a negative regulator of p53, mot-2 prevents p53 from translocating to the nucleus and functionally disables this tumor suppressor, which results in switching the binding preference of CBP from p53 to NF-κB. Our results expand the understanding of the carcinogenic potential of arsenite by identifying a mechanism whereby NF-κB regulates p53. Our results also illustrate how arsenite inactivates p53 to lead to neoplastic transformation of HELF cells.
